# Economic evaluation: a reader’s guide to studies of cost-effectiveness

**DOI:** 10.1186/s40945-022-00154-1

**Published:** 2022-12-15

**Authors:** J. Haxby Abbott, Ross Wilson, Yana Pryymachenko, Saurab Sharma, Anupa Pathak, Jason Y. Y. Chua

**Affiliations:** 1 Centre for Musculoskeletal Outcomes Research, Otago Medical School, University of Otago, Dunedin, New Zealand; 2 Health Economist & Research Fellow, Otago Medical School, Centre for Musculoskeletal Outcomes Research, University of Otago, Dunedin, New Zealand; 3 Health Economist & Postdoctoral Fellow, Otago Medical School, Centre for Musculoskeletal Outcomes Research, University of Otago, Dunedin, New Zealand; 4 Otago Medical School, Postdoctoral Fellow, Centre for Musculoskeletal Outcomes Research, University of Otago, Dunedin, New Zealand; 5 Graduate Research Student, Otago Medical School, Centre for Musculoskeletal Outcomes Research, University of Otago, Dunedin, New Zealand

**Keywords:** Cost effectiveness, Cost-effectiveness analysis, Cost-utility analysis, Economic evaluation, Health care economics, Health economics

## Abstract

**Background:**

Understanding what an economic evaluation is, how to interpret it, and what it means for making choices in a health delivery context is necessary to contribute to decisions about healthcare resource allocation. The aim of this paper to demystify the working parts of a health economic evaluation, and explain to clinicians and clinical researchers how to read and interpret cost-effectiveness research.

**Main body:**

This primer distils key content and constructs of economic evaluation studies, and explains health economic evaluation in plain language. We use the PICOT (participant, intervention, comparison, outcome, timeframe) clinical trial framework familiar to clinicians, clinical decision-makers, and clinical researchers, who may be unfamiliar with economics, as an aide to reading and interpreting cost-effectiveness research. We provide examples, primarily of physiotherapy interventions for osteoarthritis.

**Conclusions:**

Economic evaluation studies are essential to improve decisions about allocating resources, whether those resources be your time, the capacity of your service, or the available funding across the entire healthcare system. The PICOT framework can be used to understand and interpret cost-effectiveness research.

## Background

What should we choose?

There are many health services that we can provide as a health system, organisation, or provider, but only finite resources with which to provide them. Choices must inevitably be made. How do we decide?

Economics is, essentially, the science of making choices. Health economics provides *a framework for informing decisions (choices)* based on maximising outcomes from available resources: what option(s) would provide the greatest health gain for the resources (people, time, money…) available [1]. At the core of health economics is the principle of ‘utility maximisation’^1^—that is, decisions that optimise allocative efficiency [4] across the many interventions and programmes that a health service can provide so as to achieve the optimal allocation of resources, across all potential opportunities, to achieve the best possible health outcomes – to get the greatest bang for the buck [5]. (Health delivery at the population level is, of course, not entirely that simple. Economic evaluation is not the only framework relevant to decision-makers; there are other very important considerations such as equity and distributive justice that are beyond the scope of this paper [6].)

The importance of making sound allocation decisions is exemplified when we look at the bigger picture of resource allocation across the whole of the health sector, from public health through preventive interventions, community services, primary care, hospital services, medications, surgeries, allied healthcare services, diagnostic imaging, and other health technologies: when the budget is finite, resource used on one thing means that we must forgo some other potential use of that resource. This is known as the opportunity cost [7].

As clinicians and clinical researchers we should understand the health economic evaluation framework, so that we can make and influence decisions about health resource allocation – whether those decisions occur at the person level, where providers have a responsibility to ensure that health funds are used wisely [8], or up at the system level e.g. advocating for healthcare provision to a whole patient population or for funding policy regarding professional provider groups.

Understanding what an economic evaluation is, how to interpret it, and what it means for making choices in a health system is necessary to contribute to decisions about health resource allocation. Existing articles for clinician and clinical researcher audiences focus either on explaining health economics as a distinct discipline (like it were a foreign country with unfamiliar customs) or on critical appraisal and reporting standards (here’s a map and some common phrases, off you go!). This primer will instead explain how to read and interpret cost-effectiveness research by approaching health economic evaluation as an extension of the familiar clinical trial framework. We will demystify and explain in plain language the working parts of a health economic evaluation, recommend some further reading (for those interested), and provide some examples from the physiotherapy literature. The authors are end-users of clinical literature, including clinician researchers ([blinded]), postgraduate research trainees ([blinded]), health practitioners ([blinded]), health policy advisors ([blinded]), health practitioners from low-income countries ([blinded]), and readers of English as a second language ([blinded]), as well as two health economists and a clinical epidemiologist with applied health economics research experience; we have distilled the content, concepts, interpretation and implications conveyed in this primer for clinician readers.

## A reader’s guide to studies of cost-effectiveness

### A framework for understanding economic evaluations

A full economic evaluation compares the costs and the health outcomes of two or more treatment approaches. (Partial economic evaluations either make no comparison, or describe only the costs or the consequences of a treatment or approach [9] – this primer focuses on full economic evaluations.) Full economic evaluations can be thought of just like a randomized clinical trial (RCT): they estimate the incremental effects of choosing one intervention or treatment over another. Indeed, the best quality cost-effectiveness evidence comes from economic evaluations conducted within (parallel to) an RCT, making use of the unique ability of an RCT to identify the causal effects of interventions. These are known as *trial-based evaluations*. These in turn can inform *model-based evaluations*, in which decision-analytic or state-transition computer simulation models are used to evaluate scenarios too broad or complex for a single trial[10] [see Table 1].

**Table 1:** The two basic study designs of economic evaluations

Trial-based evaluations	Resource use and health-related quality of life data are recorded for all participants over the duration of a clinical trial; Cost-effectiveness of the treatment relative to control is estimated in the specific context of the trial in which the economic evaluation is nested
Model-based evaluations	Data from multiple sources, such as randomised controlled trials, observational studies, epidemiological data, and administrative records, are combined; Mathematical models are used to estimate costs, effects, and cost-effectiveness of hypothetical (modelled) scenarios; Useful when no single trial has collected all of the required data, when results from one context are to be applied in a different setting or population, or to evaluate more complex scenarios or long-term outcomes beyond the feasible scope of a randomised trial

And just like an RCT, the PICOT framework – Population, Intervention, Comparison, Outcome, and Timeframe – is an excellent aid to understanding what an economic evaluation is telling us [11, 12]. To apply that framework to economic evaluations requires only a few minor extensions of each of the PICOT criteria (Table 2). Both trial-based and model-based economic evaluations can be interpreted using the basic PICOT framework.

**Table 2:** The PICOT Framework, with extensions helpful to interpreting the findings of economic evaluations

Population	Are the patients studied like the patients I see? Are the results reported on the basis of per-person treated, per-capita (of the whole population), per x,000 people, for a whole national/state population, …?
Intervention	The same meaning as in the interpretation of a RCT
Comparison	Is the comparison genuinely a real-world alternative (i.e. what a typical patient in the study setting would otherwise get)? If not, it is difficult for a health service decision-maker to interpret what the results mean.
Outcome	Costs: What is the perspective for counting the costs? Is it strictly the payer; the whole health system; are *all* health costs counted or just ones directly attributable to the disease/condition; does it include costs borne by the patient; does it include wider societal costs, such as welfare benefit payments and productivity? How wide is the net cast? What is the ‘effects’ outcome (e.g. QALYs, deaths, responders, units of an OM? Be aware of what form are the results presented, so you can make sense of the numbers.
Timeframe	How long after intervention are the costs and effects being measured? This is known as the time horizon. The longer the time horizon, the greater the time available to accrue possible costs and effects.

Notes: *PICOT* Population Intervention Comparison Outcome, and Timeframe, R*CT* randomized clinical trial, *QALYs* quality-adjusted life-years, OM = outcome measure

### Understanding economic evaluations using the PICOT framework

#### Population

In an RCT, the *Population* refers to the patient population, or what kind of person or group of people were included in the study, the extent of inclusion and exclusion criteria, the setting from which they were recruited and in which they received the interventions – and thus to whom the results can be generalized. This is also true of economic evaluations, but in addition to that take note of the population size when interpreting the results – i.e. what is the size and scale of the population that the results are reporting on: do the authors report the total costs and effects on a per-person basis, the sum of 100 people, 100,000 people, per capita adjusted for distribution across the whole national population, or the sum for whole national population? There is a lot of variation in the way results are reported in the literature, especially for modeling studies; making sense across different reports can take some figuring out.

#### Intervention & Comparison

The *Intervention* naturally has the same meaning for both RCTs and economic evaluations, but in an economic evaluation the *Comparison* group has far-reaching consequences for the interpretation. Some RCTs focus on the incremental effects of adding an intervention on top of background care (for example, the effects of interventions provided in a physiotherapy programme provided in addition to usual medical care over-and-above those of the ‘control’ comparison group receiving only usual medical care [13]) while others focus on comparing one intervention to another (for example, comparing an exercise therapy intervention alone to exercise therapy plus manual therapy; or comparing home exercise alone to class-based exercise plus home exercise [14, 15] respectively). In either case, the between-group comparison reveals the incremental effect of the more-effective treatment over the less-effective alternative. Similarly, economic evaluations are (or should be) *incremental*, i.e. the aim of the research design is to reveal the *net* effect of the *Intervention*, over and above any effect attributable to the *Comparison*. [16] Remember: health economics is *a framework for informing decisions;* so choice of comparison matters, and must be interpretable to a decision-maker.

In almost all circumstances, the health resources available for a patient population are already being allocated to *something*. That something is the status quo: it is what is currently being delivered to the population of interest. As the question being answered by an economic evaluation is whether a net gain in value for money can be achieved by investing in the *Intervention* rather than the *Comparison*, it follows that often the most sensible, ideal comparison for useful real-world interpretation in an economic evaluation is current usual care – i.e. either background care (the real-world status quo) [13] or an established effective therapy that an intervention could be *added to *[14] – or whatever current or standard best-practice care a new intervention would potentially *replace*. [17] For example in [13, 14] the intervention is tested *in addition* to usual care or best-practice care, respectively; while [18, 19] test whether an intervention might *replace* the comparison. These are two quite different decision contexts, that the trial designer and reader must appreciate. Comparison with something artificial, that is not a normal part of health delivery – like a sham procedure that takes time and resources and has contextual effects – does not fall into either a ‘*added to’* or a ‘*replace*’ decision category, so is very difficult for a health service decision-maker to interpret, in terms of what the effects would be of implementing the intervention in their own setting, because the results do not speak directly any real-world alternative.

#### Outcome

The *Outcome* is perhaps the biggest difference between an economic evaluation and an RCT. In an RCT, there is usually one primary outcome – such as a patient-reported outcome measure, clinical measurement, or physical performance test. In an economic evaluation, there are two outcomes: the costs (the net investment) and the effects (the consequences resulting from that investment) [17]. These are typically reported as a ratio of one over the other, such as cost per quality-adjusted life-year (QALY) gained. In practice, these two outcomes can be subsequently combined by valuing the *effects* in the same units as the *costs* – i.e. monetary value – but the starting place is always units of cost and units of effect.

##### The Costs Outcome

Thinking that the only relevant costs are those of directly delivering the intervention is a common mistake. There are a broad range of consequences that flow from the decision to invest in a treatment or programme, and each economic evaluation must choose and define how broadly costs are counted [17]. This defines the *perspective* of the analysis. Narrowest is the *payer perspective*, in which the only costs considered are what a payer (typically an insurance company or government reimbursement agency) pays to the provider for delivering the treatment or programme. Broader is the *health system perspective*, which also counts up other healthcare utilisation downstream from the decision to invest (e.g. costs borne by other parts or payers in the health system, additional costs from dealing with adverse events, and cost-savings from reduced healthcare utilisation in other areas such as imaging, specialist consultations, surgeries, or medication consumption). Broadest is the *societal perspective*, in which wider, non-health system financial consequences are tallied up, such as the out-of-pocket costs borne by patients, cost burdens to family and caregivers such as time off work to care for the ill patient or provide them transport, government-paid social benefits such as disability benefits or unemployment benefits, and productivity losses through sick leave and other time off work, reduced duties or ‘presenteeism’. Other perspectives exist [3]. Clearly, the perspective chosen will make a big difference to the cost side of the cost-effectiveness equation, so understanding which perspective is being used is crucial to interpreting the results and comparing results across studies. There is no consensus regarding what perspective is most appropriate to report; often studies will report two or more perspectives to aid comparability among studies.

The two main methods of measuring costs are by patient-reported instruments, such as a log-book or a questionnaire, or by extracting data from administrative databases, or both [20]. Calculating the cost of providing the intervention itself can take a narrow (e.g. a payer’s set price) or broader approach (e.g. utilisation of plant, such as the space used in a building, power usage, clinical and administrative staff costs, and/or overheads, profit margin, etc.), but it must be calculated the same way for both the intervention and the comparison.

##### The Effects Outcome

There are 4 main types of economic evaluation, according to how effects are captured: cost–benefit analysis; cost-effectiveness analysis; cost-utility analysis (CUA; actually just a sub-set of cost-effectiveness analysis); and cost-minimisation analysis (Table 3). [21]. Each has its useful place, but CUA has the advantage of a common unit of effect (QALYs, or less commonly DALYs, disability-adjusted life years) that is comparable across diseases and settings, and thus are the most commonly seen in the clinical literature; this article will focus on CUAs.

**Table 3:** The different types of economic evaluation

Cost–benefit analysis (CBA)	Effects are measured in monetary units
Cost-effectiveness analysis (CEA)^i^	Effects are measured in any other unit of effect, e.g. deaths averted, jobs saved, treatment responders, units of a patient-reported outcome measure, …
Cost-utility analysis (CUA)	Effects are measured in QALYs (or less commonly DALYs), which are utilities summed over time
Cost-minimisation analysis (CMA)	Effects are not considered, just costs alone

^i^CEA is also known as cost-consequences analysis

In a CUA, the basic unit of health effect is known as *Utility*. [22]. Utility is typically derived from health states captured using a quality of life survey instrument such as the SF-12, SF-36, EQ-5D, HUI3, AQol-8D, 15D, or QWB.^2^ These are then scored using a *value set*, which assigns to each health state a *utility* value estimated from population *health state preferences* research. [21]. Utility values are essentially the average person’s preference for a given health state relative to a scale from death (zero) to perfect health (1). (Utility can theoretically have a negative value, for health states considered worse than death). These numerically expressed preferences are derived from studies (*health state preferences* research) in which people make tradeoffs between different health levels and life expectancy (sets of many questions like “would you rather live 10 years with poor health or only 2 years with excellent health”). So utility is just like the score on any other patient-reported outcome measure – it reflects how the patient feels about his or her health at any particular point in time.

**Fig. 1:**
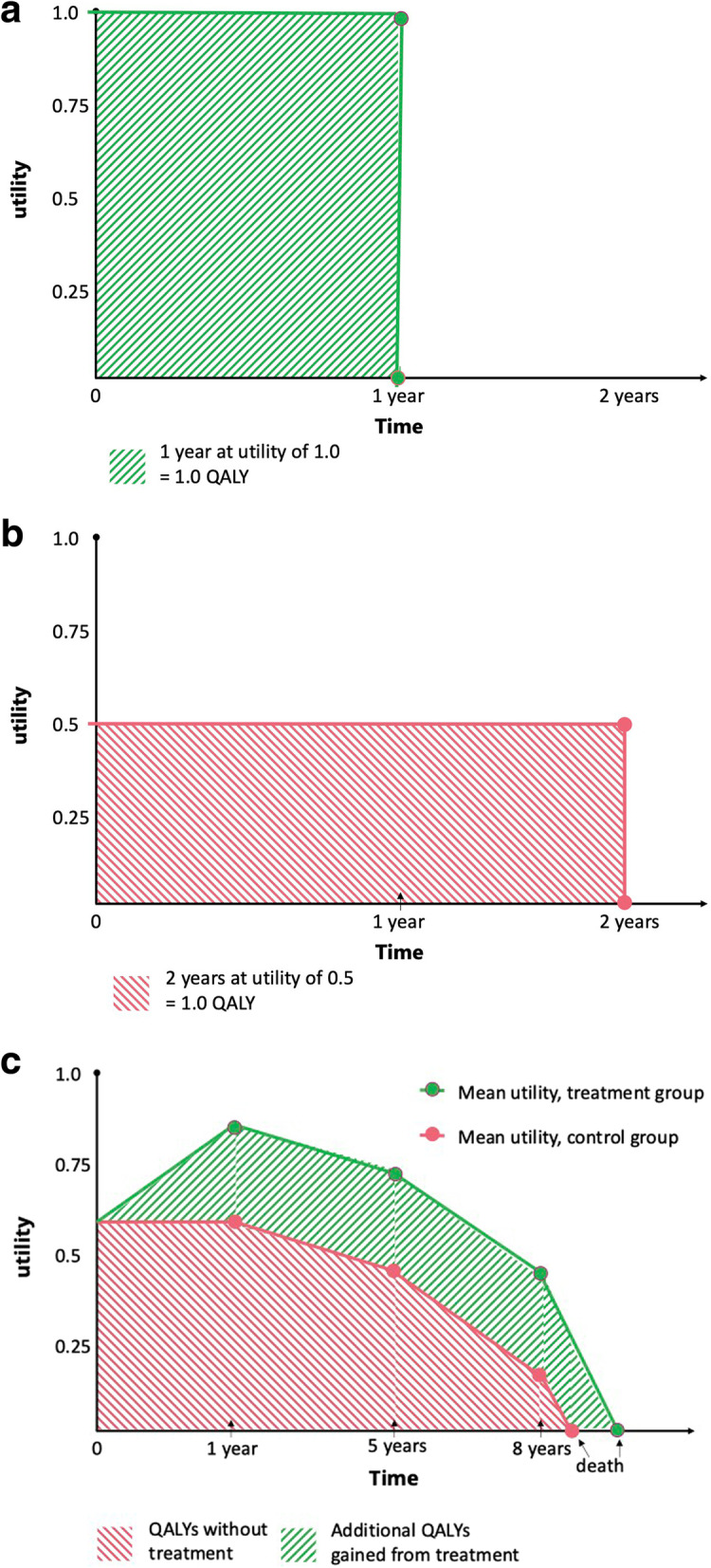
How utilities are tallied up to calculate QALYs. 1a & 1b: QALYs are calculated 2 dimensionally, using an ‘area under the curve’ method, so 1 year at full health is 1 QALY, and 2 years at utility of 0.5 is also 1 QALY. 1c illustrates the ‘area under the curve’ in a hypothetical study that followed people until death, with interim follow-up data collection points at 1, 5, and 8 years. We see that the control group experienced 3.95 QALYs and the treatment group experienced 5.95 QALYs, so the QALY gain from treatment (the area in green) is approximately 2.0 QALYs

The utilities are then summed over the time spent in each given health state to calculate quality-adjusted life years, or QALYs. One QALY is equivalent to one year spent in perfect health (or at least self-perceived full health). For example a person who lives one year in full health experiences 1 QALY in that time (Fig. 1a). A person with chronic disease in which they experience utility of 0.5, will experience ½ a QALY in one year, and 1 QALY over two years. QALYs are thus a measure of the total amount of (quality-adjusted) health experienced by an individual over a period of time; so even though utility is measured on a scale from 0 to 1, the QALYs reported in a given study can range from 0 (or potentially negative) to the length of follow-up (in years). The above describes utility informed by quality of life (the basis of quality-adjusted life years, QALYs); utility can also be defined in terms of disability (the basis of disability-adjusted life years, DALYs), and used in economic evaluation in terms of, e.g. cost per DALY averted. These are not interchangeable, but the essential application in a CUA is the same, and give generally consistent results. [23, 24].

#### Timeframe

Measuring the costs and effects outcomes this way, it is clear that the longer the timeframe, the greater the time available for possible the costs and effects to accumulate. *Time horizon* is therefore crucial to the interpretation of an economic evaluation: if the intervention has very large up-front costs and a very long period of effect (joint replacement surgery, for example) [25], a short (e.g. six-month) time horizon will not show very favourable cost-effectiveness, whereas a long time horizon (e.g. 15 years, or lifetime) is much more likely to, because the initial cost is divided by the total accrued effects. [25] Of course, we would need convincing evidence of long-lasting effects (for example 5-yr follow-up of a clinical trial that demonstrated incremental effects of a treatment compared with a real-world comparator [26]), and also expect that any downstream costs (such as expensive or fatal adverse events) are captured, otherwise the results will be distorted.

## Interpreting the results of an economic evaluation study

### How the *Outcomes* are analysed and presented

The form of results that many readers will be most familiar with from cost-effectiveness studies is the *incremental cost-effectiveness ratio*, or ICER. This is typically the net input costs (in monetary units) to achieve each unit of effect; for CUAs that unit is QALYs.^3^

**Fig. 2:**
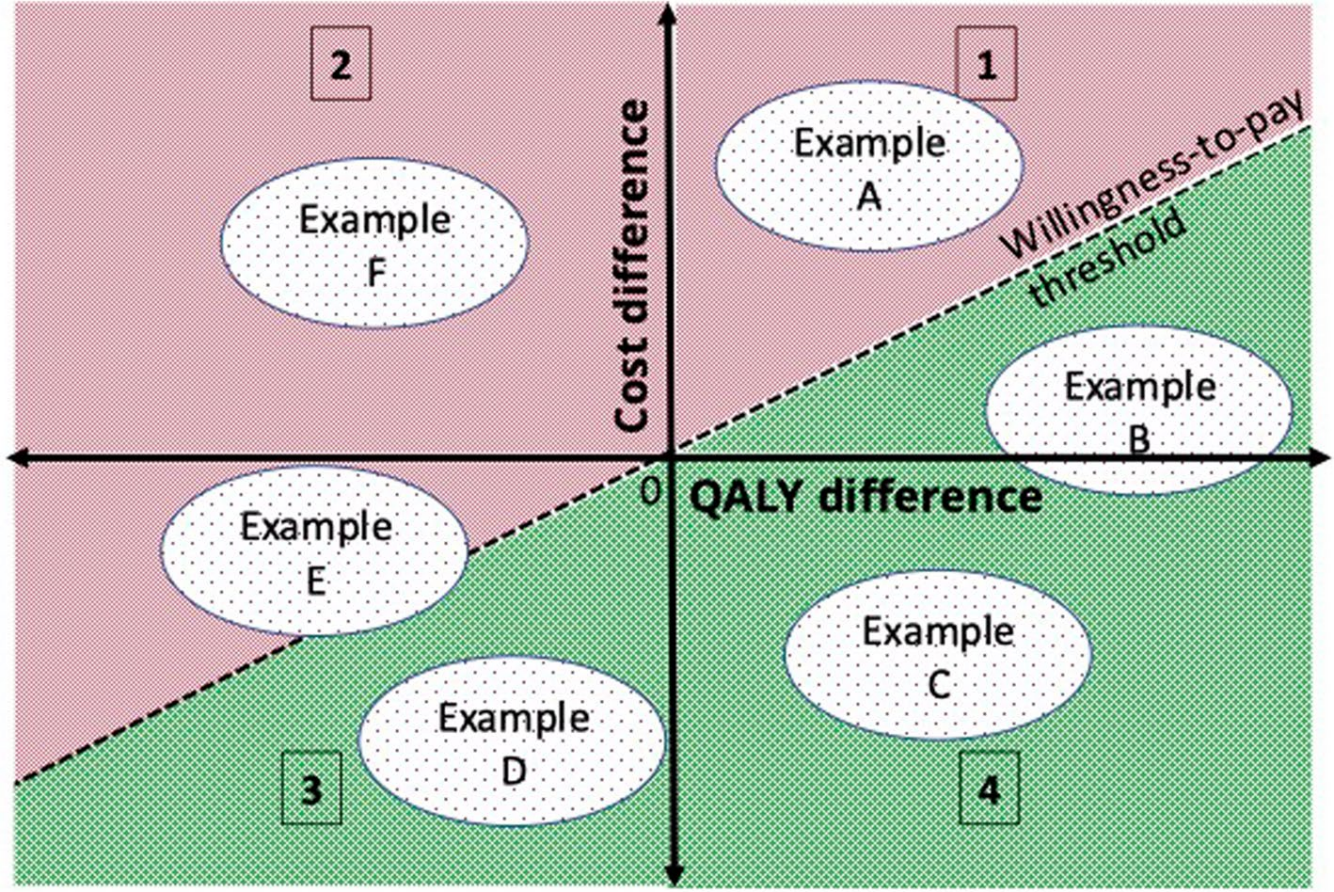
The cost-effectiveness plane and how to interpret it. Notes: The cost effectiveness plane has 2 axes that illustrate the incremental difference between the intervention group(s) and the comparator group. Difference in effects (typically in QALYs) on the x axis, and difference in costs on the y axis. Quadrant 1: costs more, and greater effects; Quadrant 2: costs more and is less effective (dominated); Quadrant 3: lower costs, but less effective; Quadrant 4: lower costs and greater effects (dominant). Example A: not cost-effective, because the cost-effectiveness is greater than the willingness-to-pay threshold; Example B: is cost-effective, because despite higher costs, it is lower than the willingness-to-pay threshold; Example C: a no-brainer – gets superior effects at lower costs; Example D: is cost-effective if you’re willing to accept inferior effects to save costs; Example E: is not cost-effective, because despite lower costs the inferior effects are above the threshold tolerable (but with some uncertainty, as the uncertainty interval, indicated by the outer cloud, crosses the WTP threshold); Example F: Fail. More costly and less effective. Also a no-brainer

It seems, on the face of it, logical that a negative ICER would be a good thing, as it would imply a cost-saving paired with an effect gain, but that assumption can be a trap. [27]. As the ICER is a ratio, it can become negative if either the numerator (net input cost) or the denominator (QALYs) is negative. From the ratio alone we cannot tell which. So an ICER is best interpreted graphically, on a cost-effectiveness plane (Fig. 2). [27]. This is a graph with 2 axes – typically costs on the y-axis and effects on the x-axis. Naturally, this results in four quadrants: 1) more costly and more effective; 2) more costly but less effective; 3) less costly but less effective; and 4) less costly and more effective. Clearly, the bottom right quadrant (4) looks like the “no brainer” choice, where the intervention returns a positive health gain at a cost saving – health economists call this dominant [17] – but there may be other good reasons not to choose this, for example where it may worsen already problematic inequity [3]. A result in quadrant 4 gives a negative ICER. Note well, however, that an ICER in quadrant 2 (more costly and less effective; known as dominated) will also be a negative number, but a far less desirable choice. This ambiguity is a potential pitfall for interpreting ICERs [17].

To interpret ICERs in Quadrant 1 (more costly, but also more effective), we need to know just how much cost we are willing to bear in order to get one unit of effect. This is known as the *willingness-to-pay* (often abbreviated WTP). The willingness-to-pay can be drawn on the cost-effectiveness plane as a diagonal line, running through the origin and with the relevant cost value (slope) per unit of effect. The interpretation is thus: any estimated ICER that falls below and to the right of the willingness-to-pay line is considered cost-effective, and anything above and to the left is not. Once we add this line to the cost-effectiveness plane, it is evident there are 6 potential outcomes (examples A through F, Fig. 2). The further below the line, the more cost-effective the treatment or programme is.

WTP is specific to each context – e.g. a national health system may have a stated or widely-accepted willingness-to-pay threshold (US$100,000 in the USA; GBP£20,000–30,000 in the UK). [28]. When interpreting results across varying contexts a scale reference such as the World Health Organisation (WHO) thresholds of 1x, 2x, and 3 × Gross Domestic Product (GDP) per capita per year can be useful, as it normalises the result to a metric (GDP) that is common but unique to each context. The WHO guidance indicates that a cost-per-QALY of less than 1 × GDP is considered highly cost-effective, between 1 × and 2 × GDP cost-effective, and more than 3 × GDP not cost-effective, but caution these rough guides are not intended to be used in country-level decision-making. [29]. Recent evidence suggests lower thresholds (often around 0.5 × GDP [30]) may also be valuable to identify more highly cost-effective interventions – better reflecting the ‘opportunity cost’, or what is given up by not using the resources on something else.

The willingness-to-pay allows another common form of results to be calculated: the *incremental net monetary benefit* (INMB, or NMB). These are more straightforward to interpret than ICERs, and avoid the ambiguity of what a positive or negative ICER means. The NMB is a unit estimate, calculated by the product of incremental effects (e.g. QALYs gained) and willingness-to-pay, minus incremental costs. For example, assuming a willingness-to-pay of €30,000 per QALY, if an intervention results in an average gain of 0.5 QALYs and has net costs of €10,000 per patient, the NMB would be (0.5 × €30,000) = €15,000 (the amount we would potentially be willing to pay for this health gain) − €10,000 (what it actually cost) = €5,000. A positive NMB means that the treatment is cost-effective at the given willingness-to-pay threshold, and thus a worthwhile investment compared to the comparator – although of course one must then consider whether or not it is a *better* investment than other opportunities that may be available.

#### Uncertainty

As with any form of statistical analysis, the results of economic evaluation are uncertain, and it is important to consider not only what the best estimate of cost-effectiveness is, but also how confident we can be that this is true. Cost data are typically widely varying and highly skewed. The type of classical inference testing used in RCTs would require much larger sample sizes to reach statistical significance – but the economic evaluations should not be interpreted using such statistical significance testing. [31, 32]. Instead, health economists advise that the point estimates (means) of the effects and costs should be used in the primary analysis. The purpose of economic evaluations is to inform decision-making, so economists separate the results useful for decision-making (the mean estimate) from the results useful to inform whether more information is required (the uncertainty interval), and argue the former should comprise the primary analysis, because failing to make a decision can and will result in measurable costs (both health and economic). [31, 32].

**Fig. 3:**
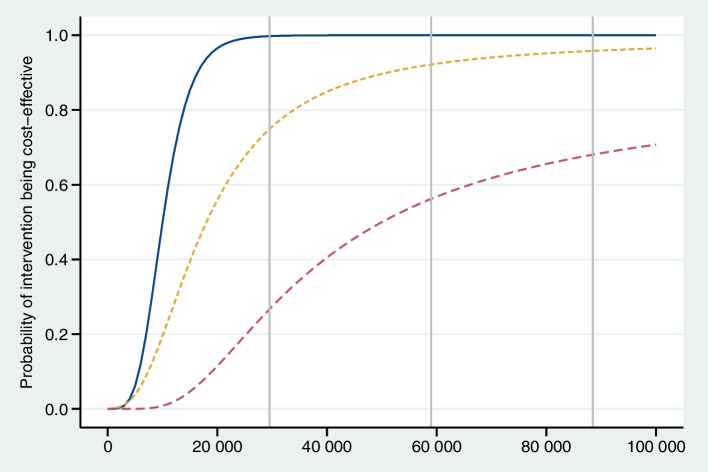
The cost-effectiveness acceptability curve. Notes: The axes of cost-effectiveness acceptability curve (CEAC) represent the probability that the intervention would be cost-effective relative to the comparator (y axis) at any given willingness to pay per QALY gained (x axis). The 3 vertical lines illustrate WTP thresholds, in this case of 1x, 2 × and 3 × GDP per capita per year. This CEAC indicates that treatment A (solid blue) would be practically certain to be cost effective at a WTP of 1 × GDP; treatment B (orange dashed) would be around 75% likely to be cost effective at a WTP of 1 × GDP; and treatment C (blue dashed) appears to be unlikely (less than 30% likely) to be cost effective at a WTP of 1 × GDP, compared with the no-treatment comparator

Uncertainty can come from not having enough data. To aid interpretation, statistical uncertainty of patient-level data can be shown as a cloud around the point estimate on the cost-effectiveness plane (Fig. 2) and/or confidence intervals around estimates in the results tables. Inaccuracy can arise from data that is not adequately representative or accurate due to some form of bias. Sensitivity analyses are often conducted and presented to show what would be the results if the range or value of some key data inputs – such as the costs of the intervention or other cost input, the treatment effects, or the patient population mix – were systematically greater or lesser than what has been assumed in the primary analysis. To aid decision-making, the *cost-effectiveness acceptability curve* (CEAC) is often reported [9]. The CEAC visualises the *probability* that delivering the intervention or programme will be cost-effective across a range of willingness-to-pay thresholds (Fig. 3). The *probability* represents 1—*P* for a 1-sided hypothesis test for a difference between the intervention and comparison [9]. If the intervention is estimated to have a positive *effect*, the CEAC will increase with higher willingness-to-pay thresholds – the more we are willing to pay for the health gains, the more likely it is that the intervention will be considered cost-effective. Relevant willingness-to-pay levels – for example the 0.5x, 1x, 2x, and 3 × GDP thresholds – can be drawn as vertical lines to aid interpretation.

### Generalisability and Quality

Just as you might ask yourself PICOT questions after reading a RCT paper: “do these results apply to the kind of patients I see? What does this mean for my context?” (*Population* questions), the same framework of questions apply for an economic evaluation paper. Key among these are: are the *Intervention* effects data from a credible, high-quality source; is the *Comparison* one that would actually be delivered in the real world (ideally what actually *is* being delivered currently); what *perspective* has been taken for the costs *Outcome*; and is the *Time horizon* appropriate to the intervention and context. A CUA using a sham intervention comparison, for example, makes little sense, because there is not a clear ‘*added to’* or ‘*replace*’ interpretation. The results do not speak directly any real-world alternative. Thus, CUA (Table 3) is not recommended for sham or placebo-controlled trials of complex, non-drug interventions such as physiotherapy interventions (for example [33]) Instead, CEA is an appropriate choice because it provides an indication of the financial resources that might be required to gain each additional unit of effect caused by the intervention, without implying a real-world “*added to’* or ‘*replace*’ interpretation.

How might the results translate from the health system where the study was conducted to the context and patient *Population* you work in? Greenhalgh provides a useful list of “ten questions to ask about an economic analysis” (Supplement 1). [34, 35]. In addition, a quality appraisal checklist can be useful to guide you through critical appraisal of the methods underlying the study (e.g. the CHEC list [36, 37]), as well as the RCT or (for a modelling study) systematic review from which the data came, [38] or the decision model that produced the results. [10, 39].

## Making choices: how to use the results of an economic evaluation

Choosing wisely is important to reduce waste and harm from unnecessary and low-value health services [8]. As more and more health technologies, treatment options and services become available to us over time, and population health needs are growing, economic evaluations must play an increasingly important role. Systematic reviews of cost-effectiveness research are now appearing [40], as are modeling studies of multiple competing treatment options. [41]. Knowledge is power, so it is crucial, as clinicians, clinical researchers, and patient advocates, to empower ourselves to recognise high-value care by having at least a passing familiarity with the health economic evaluation framework.

But doesn’t adopting new innovations, even cost-effective ones, always cost more? No. In your service or your clinical practice, you can deliver better health outcomes for a fixed budget by choosing to use your finite resources on more cost-effective interventions or programmes, and disinvesting in low-value, cost-ineffective ones. For example, disinvesting in more costly routine individualized and supervised outpatient physical therapy after total knee arthroplasty (TKA) in favour of less resource-intensive home-based exercise interventions, or de-implementing use of continuous passive motion (CPM) machines after TKA could result in similar effects for much lower costs, freeing up resources to invest in better value interventions.[42, 43]. In this way, the net cost from *your* perspective can be zero, but result in greater health gains. Further, if you choose wisely the net cost from the health system or societal perspective may actually be less than zero, if the new intervention (and the better health it delivers) results in lower downstream healthcare consumption and productivity losses, fewer adverse events or longer life (as, for example, was seen with individually supervised exercise therapy in addition to usual care for people with hip or knee osteoarthritis [44]).

As a clinician or service leader, knowledge of the health economic evaluation framework is useful in an advocacy role, making a case to the planning & funding decision-makers for new services to serve a patient population. An example of this comes from our experience in the orthopaedic service at a public hospital serving a main city and large surrounding region in New Zealand. [45]. Due to limitations of funding and capacity, joint replacement surgery is rationed by a prioritisation system based on disease severity. General practitioners were referring patients with osteoarthritis for an orthopaedic consultation, as they felt joint replacement surgery was the appropriate next treatment. However, demand outstripped supply, so only the most severe cases were able to be offered appointments. The rest were turned away, back to the GP, resulting in a growing unmet need. As a clinical researcher active in conducting RCTs in association with the local service^17−22^, one of us ([blinded]) proposed a new clinic to serve this unmet need. Others had proposed a similar thing before and got nowhere, but this time, recognising that the language of funding decision-makers is dollars and sense, we came with a business case based on real, local RCT data [13] with a full parallel economic evaluation demonstrating not just cost-effectiveness, but cost savings. [46]. QALY gains were greater, and the cost savings attributable to the intervention came not only from reduced health system costs (less medications, imaging, doctor visits, etc.) but also societal perspective costs such as out-of-pocket costs to the patient and family, and substantial reduction in productivity losses. The cost savings more than recouped the cost of providing the intervention. The results were robust to uncertainty analyses, and persisted at both one- and two-years follow-up. [44, 46]. The door opened, a partnership in funding, developing and implementing the new service was entered, and one result was a 90% reduction in unmet need. [45]. People previously turned away were being seen, and receiving high-value care. [41]. This illustrates the opportunities for clinicians and clinical researchers to use the health economic evaluation framework in an advocacy role for patient populations at the service delivery level.

## Conclusion

For a clinician or clinical researcher, economic evaluation studies may seem complex; we hope this primer has helped demonstrate that the interpretation of these studies is not complicated; rather, it is comparable to interpreting a RCT. While accepting that clinical decision-making and policy-making are complex processes that must take into account many other factors, studies of cost-effectiveness are essential to improve decisions about allocating resources, whether those resources be your time, the capacity of your service, or the available funding across the entire healthcare system. We have outlined how the familiar PICOT framework – Population, Intervention, Comparison, Outcome, and Timeframe – is useful to clinicians and clinical researchers reading studies of cost-effectiveness, and interpreting the meaning and generalisibility of economic evaluations.

## Data Availability

The data supporting our findings can be found in the literature cited in the paper, listed in the reference list, and additional references by request to the corresponding author.

## Abbreviations

CBA: Cost-benefit analysis
CEA: Cost-effectiveness analysis
CEAC: Cost-effectiveness acceptability curve
CMA: Cost-minimisation analysis
CUA: Cost-utility analysis
DALY: Disability-adjusted life year
GDP: Gross Domestic Product
ICER: Incremental cost-effectiveness ratio
INMB or INB: Incremental net monetary benefit
OM: Outcome measure
PICOT: Participant, intervention, comparison, outcome, timeframe
QALYs: Quality-adjusted life-years
RCT: Randomized clinical trial
WHO: World Health Organisation
WTP: Willingness-to-pay

## Footnotes

1 A foundational principal in economics is the concept of ‘the rational person’ who makes choices on the principle of *utility maximisation* (although some behavioural economists have been studying irrational choices, even before 2016). Health economics largely extends this neoclassical economic theory to *welfarism* (or more accurately and specifically, extra-welfarism) [2, 3].

2 Short Form 36 item questionnaire; Short Form 12 item questionnaire; Health Utility Instrument; 8 dimension Assessment of Quality of Life instrument; 15 Dimension health-related quality of life instrument; Quality of Well-Being scale.

3 Such a ratio should then be called ICUR, for Incremental Cost Utility Ratio, but often isn’t, so we will continue using the more commonly-used acronym ICER.

## Glossary

Allocative efficiency: the optimal allocation of resources, across all potential opportunities, to achieve the best possible health outcomes.
Cost-benefit analysis (CBA): an economic analysis in which the effects are measured in monetary units.
Cost-consequences analysis (CCA): another name for cost-effectiveness analysis.
Cost-effectiveness analysis (CEA): and economic evaluation in which effects are measured in any unit of effect other than monetary units, e.g. deaths averted, jobs saved, treatment responders, units of a patient-reported outcome measure, …etc.
Cost-effectiveness acceptability curve (CEAC): a graph that illustrates the probability that delivering the intervention or programme will be cost-effective on the y axis, against the cost-per-unit of effect on the x-axis.
Cost-utility analysis (CUA): A type of CEA where the effects are measured in QALYs, which are utilities summed over time.
Cost-minimisation analysis (CMA): Effects are not considered, just costs alone Economic analysis: a structure of a statistical analysis of the costs component of an economic evaluation
Economic evaluation: a study that aims to investigate the value of something in terms of both costs and effects.
Equity: the absence of avoidable gaps in health outcomes or health services between groups of differing levels of socio-demographics
Health state preferences: see ‘Utility’
Incremental cost-effectiveness ratio (ICER): a metric for describing the net input costs, in monetary units, to achieve each unit of effect
Incremental cost-utility ratio (ICUR): a particular kind of ICER in which the unit of effects is utility (generally in QALYs).
Incremental net monetary benefit: (INMB, or NMB)
Opportunity cost: resource used on one thing means that we must forgo the benefits of some other potential use of that resource.
Perspective: Defines what costs will be included in an economic evaluation.
Quality-adjusted life year (QALY): a generic measure of disease burden that accounts for both the quality and the quantity of life lived; i.e. utilities summed over time.
Utility: a measure of the preference or value that a person (or group) has for a particular health state in comparison to all potential health states, typically expressed as a number between 0 (representing death) and 1 (perfect health), but can have a negative value.
Willingness-to-pay (WTP): how much net cost we are willing to bear in order to get one unit of effect.
